# Advances in Artificial Intelligence for Plant Research

**DOI:** 10.3390/plants15010142

**Published:** 2026-01-03

**Authors:** Guoxiong Zhou, Liujun Li, Xiaoyulong Chen

**Affiliations:** 1 College of Computer & Information Engineering, Central South University of Forestry and Technology, Changsha 410004, China; 2 Agricultural and Life Sciences, Department of Soil and Water Systems, University of Idaho, Moscow, ID 83844, USA; johnnylst2@gmail.com; 3 Key Laboratory of Karst Geological Resources and Environment, Guizhou University, Guiyang 550025, China; chenxiaoyulong@163.com

## Editorial on Research Topic

### Deep Learning in Plant Science: Advancements, Applications, and Future Directions

Plants are fundamental for global food security, ecological balance, and sustainable development. Challenges such as crop disease outbreaks, inefficient phenotype monitoring, labor-intensive harvesting, and limited resource utilization have long constrained the productivity and resilience of plant-related systems. Traditional plant science research and agricultural management methods rely heavily on manual operations, which are time-consuming, labor-intensive, and prone to subjective biases—rendering them increasingly incompatible with the demands of large-scale, high-precision modern agriculture and ecological conservation.

In recent years, deep learning has emerged as a transformative technology in computer vision, data analytics, and intelligent control. By automatically extracting complex, hierarchical features from multi-source data (including RGB images, spectral data, depth data, and point clouds), deep learning significantly improves the efficiency, accuracy, and scalability of plant-related tasks. It minimizes human intervention, reduces misjudgment risks, and enables real-time decision making—addressing key issues that beset traditional methods. However, critical challenges remain: data imbalance across plant species and growth stages, environmental interference (e.g., lighting variations, occlusion, noise), poor cross-scenario generalization of models, and difficulties in deploying complex algorithms on edge devices (e.g., agricultural robots, UAVs). These barriers hinder the widespread adoption of deep learning in plant science, creating an urgent need for innovative algorithms, optimized model architectures, and practical application frameworks.

This Research Topic aims to cover the latest advancements in deep learning applications in plant science, providing a comprehensive overview of technical breakthroughs and practical implementations. The focus of this review encompasses six core directions: (1) plant disease identification and classification, (2) plant phenotype parameter estimation (e.g., biomass, leaf area, growth stage), (3) plant organ recognition (e.g., pollen, flowers, panicles), (4) intelligent robotic harvesting technologies, (5) multi-modal data fusion for enhanced model robustness, and (6) lightweight model design and edge deployment optimization.

Yu et al. (2024) [1] proposed AIpollen, a pollen identification system based on convolutional neural networks. To address the time-consuming nature of traditional pollen identification approaches and their heavy reliance on professional expertise, the team adopted a fine-tuned pre-trained ResNet34 network architecture, combining it with the Adam optimizer, cross-entropy loss function, and supplementary strategies including data augmentation, learning rate decay, and early stopping. They trained the model on a dataset covering 36 pollen genera, and it achieved 97.01% accuracy on the test set alongside a 95.9% F1 score. Additionally, a user-friendly web interface was developed, enabling users to upload pollen images and instantly obtain predicted genus names, which provides a powerful tool for pollen identification in fields such as botany, ecology, and allergy research.

Taddei Dalla Torre et al. (2024) [2] developed an AI-based vision framework for the robotic harvesting of edible flowers. To tackle the labor-intensive challenges of manual picking and the need for the precise localization of flowers, the framework employs YOLOv5 for 2D flower detection, leverages the zero-shot capability of the Segmentation Anything Model (SAM) to extract 3D point cloud features, and applies Principal Component Analysis (PCA) for pose estimation. The team also established a linear regression model that correlates flower diameter with plucking point height to determine optimal cutting positions. The results demonstrated effective 2D detection, 3D localization, pose estimation, and plucking point prediction, with a single-flower processing time of approximately 1 s, offering an adaptable technical solution for the automated harvesting of diverse edible flower species.

Hou et al. (2024) [3] proposed a deep learning model based on multimodal data fusion for lettuce phenotype estimation. Targeting the inaccuracies of traditional phenotypic monitoring methods, the model integrates RGB and depth images through a dual-branch network, incorporating a Feature Rectification Module (FRM) and a Squeeze-and-Excitation Fusion (SEF) module. It also optimizes a Feature Pyramid Network (FPN) and adopts a residual structure for the phenotypic trait head. When tested on four lettuce varieties, the model achieved high precision in estimating key traits—such as an R^2^ of 0.9732 for fresh weight and 0.9739 for dry weight—and attained AP50:95 scores of 0.8881 for detection and 0.9041 for segmentation. It thus, offers a reliable approach for monitoring lettuce growth in greenhouses and determining optimal harvest timing.

Wang et al. (2025) [4] proposed VM-YOLO, a lightweight hybrid network designed for strawberry flower detection. To overcome the computational constraints of agricultural mobile equipment and the difficulty of detecting small, dense flower clusters, the study replaced YOLOv8’s backbone with a multi-branch Light C2f module and introduced VMambaNeck to obtain a global receptive field. They trained the model on a self-constructed strawberry flower dataset, and it achieved 71.4% mAP, a 22.4 ms inference speed, and only 30 million parameters, outperforming state-of-the-art algorithms such as YOLOv6, Faster R-CNN, and RetinaNet. This design makes it well suited for deployment on resource-constrained agricultural mobile platforms.

Chen et al. (2025) [5] proposed CBSNet, a specialized model for potato leaf disease classification. Aiming to solve the problems of tiny lesions, blurred edges, and noise interference in potato leaf disease images, the model integrates Channel Reconstruction Multi-Scale Convolution (CRMC), Spatial Triple Attention (STA), and the Bat–Lion Algorithm (BLA). When evaluated on a self-built dataset, CBSNet achieved an average accuracy of 92.04% and 91.58% precision, effectively extracting subtle lesion features and blurred edge information. This work provides strong technical support for large-scale potato disease prevention and control.

Wu et al. (2025) [6] proposed OE-YOLO, an improved model based on YOLOv11 for rice panicle detection. Addressing the challenges of small size, dense distribution, and diverse growth directions of rice panicles, the model adopts Oriented Bounding Boxes (OBBs), replaces the backbone with EfficientNetV2, and introduces the C3k2_DConv module enhanced by dynamic convolution. Tested on datasets covering different flight heights (3 m and 10 m) and growth stages (heading and filling), OE-YOLO achieved 86.9% mAP50 with only 2.45 million parameters and 4.8 GFLOPs, outperforming models such as YOLOv8-obb and YOLOv11. It thus provides an efficient and accurate solution for counting rice panicles and predicting yield.

Xu et al. (2025) [7] published a comprehensive review on AI applications in forestry. Focusing on three core areas—resource monitoring, disaster management, and sustainability—the study systematically synthesizes 49 peer-reviewed articles published between 2019 and 2025. It highlights AI’s transformative potential in sub-meter precision forest canopy monitoring, high-recall wildfire detection, and mangrove carbon sequestration optimization. The review also identifies key challenges including cross-ecological model generalization, multi-source data fusion, and ethical implementation, providing actionable pathways for integrating AI into forestry management to enhance ecological security and sustainability.

Zeng et al. (2025) [8] proposed a bottom-up multi-feature fusion algorithm for individual tree segmentation (ITS) in dense rubber tree plantations using UAV-LiDAR point clouds. To address challenges such as overlapping canopies, indistinct tree apexes, and intricate branch structures, the method first extracts trunks via branch-point density variations and neighborhood directional features and, then integrates geometric, directional, and density attributes to classify core canopy points, boundary points, and overlapping regions. Disputed points are iteratively assigned based on neighborhood growth angle consistency. When tested on plots with low, medium, and high canopy closure, the algorithm achieved accuracies of 0.97, 0.98, and 0.95, respectively, with R^2^ values exceeding 0.98 for crown width and 0.97 for canopy projection area (compared to ground truth). It provides a reliable foundation for 3D tree modeling and biomass estimation in complex tropical agroforests, advancing precision forestry.

Wang et al. (2025) [9] developed ELD-YOLO, a lightweight detection framework to address issues including occlusions, small fruits, and low efficiency in complex orchard environments. The model integrates three core modules: an Edge-guided Multi-scale Dual-domain Enhancement (EMD) module for edge feature preservation, a Lightweight Task-Interactive Detection Head (LIDH) to balance accuracy and computational cost, and a Dynamic Point Sampling (Dysample) module to minimize information loss during upsampling. Trained on a 2388-image mandarin dataset, it achieved 89.7% precision, 83.7% recall, mAP@50 of 92.1%, and mAP@50:95 of 68.6%, with 15.4% fewer parameters than the YOLO11 baseline. It outperforms mainstream models (e.g., YOLOv10n, Faster R-CNN) in occluded and small-object detection, providing an efficient solution for orchard yield prediction and precision harvesting.

Xu et al. (2025) [10] proposed the Wheat Cultivation Suitability Evaluation–Agricultural Group Consensus (WCSE-AGC) framework to assess wheat stripe rust severity and cultivation suitability. To address limited expert coverage and inconsistent evaluations, the framework uses Claude 3.7 (AIGC) to simulate expert scoring via role-playing and chain-of-thought prompting. It comprises three stages: a Trust Graph Neural Network (TGNN) to complete missing trust links, a hybrid algorithm (SBO + K-means + three-way clustering) to detect overlapping expert subgroups, and two-stage optimization to balance group fairness and adjustment cost. Validated on datasets from Ethiopia, India, Turkey, and China, the framework achieves robust consensus (a final group consensus level of 0.9311) and stable rankings across parameter variations, supporting data-driven decision-making in precision agriculture.

Hou et al. (2025) [11] formulated wheat soil-borne mosaic virus (SBWMV) detection as a large-scale group decision-making (LSGDM) problem, treating each planting plot as a virtual decision maker. The framework encodes field observations into intuitionistic fuzzy numbers (IFNs) to capture uncertainty, uses a Bayesian-GCN to infer missing spatial trust values, and applies enhanced spectral clustering to group ecologically similar plots. A feedback mechanism guided by Agricultural Decision Indicator Sets–Multi-Granulation Rough Sets (ADISs-MGRS) iteratively adjusts evaluations until consensus. Validated on a U.S. Pacific Northwest dataset (2017–2018) with data augmentation, the model outperforms traditional methods in consensus efficiency (taking only 10 iterations) and ranking stability (with a Kendall coefficient of 1.097), providing interpretable and robust support for targeted SBWMV prevention and precision agriculture.

Zhang et al. (2025) [12] developed YOLOv8-BS, an optimized YOLOv8-based model for detecting sheath colors and spots of *Chimonobambusa* utilis bamboo shoots. Enhanced by data augmentation techniques such as translation, flipping, and contrast adjustment, the model integrates C2f and RepConv modules for feature extraction, SPPF and PAN for multi-scale fusion, and an anchor-free detection head with CIoU loss for precise localization. Trained on 6186 images (2062 original + 4124 augmented) from Jinfo Mountain, China, it achieved 86.8% AP (with 85.9% precision and 83.4% recall) for color detection (covering five categories) and 96.1% AP (with 90.1% precision and 92.5% recall) for spot detection (covering two categories), outperforming models such as YOLOv7, YOLOv5, YOLOX, and Faster R-CNN. It supports bamboo germplasm evaluation, genetic diversity studies, and quality grading in sustainable bamboo industries.

Deng et al. (2025) [13] proposed FCMNet, a multimodal fusion framework for tomato leaf disease identification. Addressing the limitations of single-mode identification approaches, it integrates a Fourier-Guided Attention Mechanism (FGAM) for lesion localization, Cross Vision–Language Alignment (CVLA) for image-text fusion, and a Multi-strategy Improved Coati Optimization Algorithm (MSCOA) for training optimization. On a self-built 6994-image dataset, the framework achieved 98.04% accuracy, 97.99% precision, 97.81% recall, and 97.95% F1-score, outperforming such models as ResNet50 and HCA-MFFNet. This provides technical support for intelligent agricultural disease diagnosis.

Wan et al. (2025) [14] developed FCA-STNet for cotton seedling growth prediction using RGB image sequences. To solve issues of poor spatiotemporal representation and low texture fidelity in existing models, it combines a custom STNet backbone with Adaptive Fine-Grained Channel Attention (FCA) to suppress field interferences. The model achieved an MSE of 0.0086, SSIM of 0.8339, and PSNR of 20.7011 (representing 2.27–11.20% improvements over the baseline), with over 0.8 correlation for 37 extracted phenotypic traits. This enables realistic growth prediction for precision cotton cultivation.

Yan et al. (2025) [15] proposed KBNet, a language–vision fusion framework for rice disease segmentation. Targeting the challenges of multi-scale and irregular lesions in rice disease images, it integrates Kalman Filter Enhanced KAN (KF-KAN) for multi-scale feature fusion and Boundary-Constrained PINN (BC-PINN) with physical priors. On a 1550-image dataset, it achieved 72.3% IoU and 83.9% Dice, outperforming models such as UNet (60.2% IoU) and LViT (64.0% IoU). It also generalizes well to maize and tomato datasets, supporting intelligent rice disease control.

Yuan et al. (2025) [16] developed SLMW-Net for pine wilt disease detection in UAV imagery. To address issues including complex backgrounds, subtle disease features, and prediction bias, the model includes a Self-Learning Feature Extraction Module (SFEM), MicroFeature Attention (MFAM), and Weighted Linear IoU Loss (WLIoU). Trained on the ARen dataset (750 images), it achieved 86.7% mAP@0.5 and 40.1% mAP@0.5:0.95 (representing a 2.8% mAP gain over YOLOv11) with only 3.9 M parameters. This makes it suitable for forest health monitoring applications.

Zhao et al. (2025) [17] proposed Sparse-MoE-SAM, a lightweight framework for plant disease segmentation in resource-constrained scenarios. It uses Gumbel-TopK sparse attention (which reduces complexity to O(nk)) and a dual-stage MoE decoder, enhanced by sparse ASPP. On the PlantVillage dataset, it achieved 94.2% mIoU (a 2.5% improvement over SAM) with a 23.7% reduction in FLOPs. The mobile variant (with 45.3 M parameters) enables deployment on edge devices such as Jetson Nano and smartphones, supporting precision agriculture.

Wang et al. (2025) [18] used the Biomod2 ensemble framework to predict the potential distribution of invasive *Solanum rostratum* in China. To address the need for accurate invasion risk assessment, the study compiled multi-source occurrence records and selected low-multicollinearity climate variables, integrating ten individual models within Biomod2. It adopted the committee averaging (EMca) ensemble approach to enhance prediction robustness and simulated habitat suitability under current climate conditions and four future SSP scenarios (SSP126, SSP245, SSP370, and SSP585). The method involves data screening, environmental variable optimization, model training/validation, and spatial pattern analysis (e.g., centroid shift), providing technical support for the targeted prevention and control of the invasive species.

Wu et al. (2025) [19] proposed FEWheat-YOLO, a lightweight model based on YOLOv11n for wheat spike detection in precision agriculture. To address challenges such as complex field backgrounds, small/dense targets, and resource constraints of agricultural edge devices, the study integrated four core modules. It constructed a hybrid dataset (HWHD) by combining public GWHD2021 data and self-collected field images from Xinjiang: FEMANet enhances small-target feature representation, BiAFA-FPN optimizes multi-scale feature fusion, ADown preserves structural details during downsampling, and GSCDHead reduces parameter redundancy via shared convolution. This lightweight design enables deployment on resource-limited platforms, supporting wheat yield estimation and variety selection in precision agriculture.

Li et al. (2025) [20] presented a depth imaging-based framework for tomato fruit phenotypic recognition. To address inefficient manual phenotyping and inaccurate size measurement, the study constructed a dataset of tomato fruit section images using a depth camera. It improved the SegFormer model with the MLLA linear attention mechanism (resulting in SegFormer-MLLA) for the precise segmentation of stem scars and locules and designed a Hybrid Depth Regression Model (HDRM) to optimize depth estimation. The framework integrates RGB and depth information to extract 12 phenotypic traits, supporting the precision breeding and quality evaluation of tomatoes.

In summary, these findings underscore the remarkable progress deep learning has brought to the monitoring of plant phenotypes in the digital agriculture era. In the future, these optimized multimodal models can be integrated into precision farming platforms to track crop growth traits (e.g., biomass, leaf area) in real time, allowing targeted adjustments to irrigation, fertilization, and harvest scheduling [21].

As deep learning frameworks continue to mature—with better lightweight design and cross-species generalization—we can anticipate that these tools will adopt a central role in smart breeding and crop yield optimization, delivering more precise and sustainable support for modern agricultural production.
